# Highly Efficient One-Step Protein Immobilization on Polymer Membranes Supported by Response Surface Methodology

**DOI:** 10.3389/fchem.2021.804698

**Published:** 2022-01-18

**Authors:** Martin Schmidt, Amira Abdul Latif, Andrea Prager, Roger Gläser, Agnes Schulze

**Affiliations:** ^1^ Leibniz Institute of Surface Engineering (IOM), Leipzig, Germany; ^2^ Institute of Chemical Technology, Leipzig University, Leipzig, Germany

**Keywords:** surface modification, polymer membrane, serum albumin, radiation-induced graft immobilization, electron beam, response surface methodology

## Abstract

Immobilization of proteins by covalent coupling to polymeric materials offers numerous excellent advantages for various applications, however, it is usually limited by coupling strategies, which are often too expensive or complex. In this study, an electron-beam-based process for covalent coupling of the model protein bovine serum albumin (BSA) onto polyvinylidene fluoride (PVDF) flat sheet membranes was investigated. Immobilization can be performed in a clean, fast, and continuous mode of operation without any additional chemicals involved. Using the Design of Experiments (DoE) approach, nine process factors were investigated for their influence on graft yield and homogeneity. The parameters could be reduced to only four highly significant factors: BSA concentration, impregnation method, impregnation time, and electron beam irradiation dose. Subsequently, optimization of the process was performed using the Response Surface Methodology (RSM). A one-step method was developed, resulting in a high BSA grafting yield of 955 mg m^−2^ and a relative standard deviation of 3.6%. High efficiency was demonstrated by reusing the impregnation solution five times consecutively without reducing the final BSA grafting yield. Comprehensive characterization was conducted by X-ray photoelectron spectroscopy (XPS), Fourier-transform infrared spectroscopy (FTIR), and measurements of zeta potential, contact angle and surface free energy, as well as filtration performance. In addition, mechanical properties and morphology were examined using mercury porosimetry, tensile testing, and scanning electron microscopy (SEM).

## 1 Introduction

Membrane technology is used in many modern applications such as water treatment (Pendergast and Hoek, 2011), biomedicine (Casimiro et al., 2019), pharmaceutical industry (Buonomenna and Bae, 2014), or in chromatography (Zeng and Ruckenstein, 1999). Polymer-based membrane filters dominate the multi-billion global membrane module market with a share of 92.1% (Sutherland, 2004). Polyvinylidene fluoride (PVDF) is among the most widely used materials due to excellent features such as chemical resistance, high thermal stability and mechanical strength. Thus, products prepared from PVDF are widely used in many areas (Feng et al., 2013; Kang and Cao, 2014). However, fluoropolymers have the severe disadvantage of being highly hydrophobic facilitating adsorption of organic compounds and thus, fouling (Liu et al., 2011). Surface modification of membranes has been investigated extensively in order to modify the surface hydrophilicity (Subasi and Cicek, 2017), introduce advanced functionalities (Nasef, 2004), or improve blood compatibility (Mollahosseini et al., 2020). Many methods have been studied, of which protein grafting is a widely used approach (Fang et al., 2010; Zhu et al., 2011; Liu et al., 2012; Riyasudheen et al., 2012; Wang et al., 2012; Wu et al., 2014; Akashi and Kuroda, 2015; Mayuri et al., 2020).

Bovine serum albumin (BSA) is a globular protein derived from cow serum (molecular weight M_W_ = 66.5 kDa). BSA is a commonly used biomolecule in research due to its wide availability, low cost and similarity to human serum albumin (Majorek et al., 2012). Furthermore, BSA is a carrier protein for a degradation product of hemoglobin, *i.e.*, bilirubin, with multiple binding sites per BSA molecule (Blauer and King, 1970), as well as for other substances such as drugs (Bi et al., 2009), or toxic compounds (Das et al., 2017). Hence, BSA has been immobilized and used as a functional biopolymer for various applications, *e.g.,* 1) for the improvement of blood compatibility (hemocompatibility) in blood filtration systems (Zhang et al., 2013); 2) for the reduction of hemolysis in blood storage devices (Li et al., 2014); 3) for the adsorption of bilirubin in the treatment of the liver disease hyperbilirubinemia *via* hemoperfusion (Wu et al., 2018); 4) for the removal of bacteria, dyes and proteins by water filtration membranes (Ahmad Khairuddin et al., 2018); or 5) for the generation of high-speed humidity sensors in conjugation with multiwall carbon nanotubes (Bhattacharya and Sasmal, 2016).

Immobilized proteins are used in industry and medicine due to several advantages such as convenient handling in continuous processes, prevention of product contamination, or improved stability towards changing process conditions. There are a variety of immobilization methods in order to attach proteins onto polymer surfaces, including physical adsorption, entrapment, or covalent attachment (Eş et al., 2015). The latter is the defining result of the well-established polymer surface grafting (Horie et al., 2004; Ulbricht, 2006; Szymczyk et al., 2019). Covalent immobilization is often preferred to achieve stable and high protein coverage, and to avoid delamination (Kato et al., 2003). In case of inert PVDF, however, no polar reactive sites are present, which further impairs covalent coupling. For this reason, the surface must first be activated, *e.g.*, *via* reactive reagents (Yiğitoğlu et al., 2007), plasma treatment (Kochkodan and Sharma, 2012), or ionizing radiation (Ramos-Ballesteros et al., 2019). Subsequently, a wet-chemical coupling reaction might be carried out to bind the biomolecules (Schulze et al., 2017; Schmidt et al., 2018). Nevertheless, the methods published so far have considerable drawbacks that are diametrically opposed to future industrial applications (Kang et al., 1993; Ladavière et al., 1999; Fang et al., 2009; Fang et al., 2010; Zhu et al., 2011; Liu et al., 2012; Riyasudheen et al., 2012; Wang et al., 2012; Zhang et al., 2013; Li et al., 2014; Wu et al., 2014; Akashi and Kuroda, 2015; Vitola et al., 2015; Wu et al., 2018; Mayuri et al., 2020): 1) modifications are performed in multi-step batch reactions; 2) often require several expensive or toxic coupling chemicals like acrylates, carbodiimide, or glutaraldehyde; 3) take many hours up to several days to complete; 4) involve harsh reaction conditions; or 5) are limited to selected types of polymer substrates. Therefore, protein-modified polymer materials still fall short of their true potential due to a lack of cost-effective and highly scalable manufacturing strategies.

In the last decade, a novel approach for polymer membrane surface grafting was developed (Schulze et al., 2010). Recently, a new terminology for this kind of polymer processing was proposed, *i.e.*, radiation-induced graft immobilization (RIGI) (Schmidt et al., 2021). Briefly, a membrane impregnated with an aqueous solution of a modifying compound is irradiated by an electron beam (EB) produced *via* an electron accelerator. The polymer, as well as the solutes are activated and react immediately with each other by forming covalent bonds. In contrast to the traditional radiation-induced graft polymerization (RIGP), RIGI can utilize non-vinyl compounds such as small organic stable molecules (*e.g.,* glycerin, malonic acid, or glucose) (Schulze et al., 2012), hydrophilic polymers (Schulze et al., 2013), photoactive molecules (Müller et al., 2018), enzymes (Starke et al., 2013; Jahangiri et al., 2014), or antimicrobial peptides (Reinhardt et al., 2018), instead of potentially hazardous vinyl monomers like acrylates (Suh et al., 2018), or styrenics (Gibbs and Mulligan, 1997). Since the processing is very fast and straightforward, a continuous mode of operation for industrial roll-to-roll applications is feasible (Supplementary Figure S1A in SI). Furthermore, due to the direct coupling by using solely the polymer and an aqueous solution of the modifying agent, this approach becomes highly environmentally friendly, clean and thus “green.”

Response surface methodology (RSM) is a statistical and mathematical tool introduced in 1951 (Box and Wilson, 1951), which is often used for the optimization of processes or reactions. RSM is part of a larger statistical toolbox for experimentation, *i.e.*, Design of Experiments (DoE). Compared to the conventional experimental approach, known as one-factor-at-a-time (OFAT), the DoE approach probes the entire parameter space using systematically distributed points in order to fit the measurement data with a mathematical model (Supplementary Figure S1B in SI). This multifactorial method has many advantages (Anderson and Whitcomb, 2010), including 1) reducing the number of experiments and costs; 2) rapidly generating reliable experimental data; 3) reducing numerical noise; or 4) determining effect sizes and, in particular, interactions between parameters. RSM has been applied in the field of polymer grafting in some studies (Chung et al., 2014; Madrid et al., 2017; Mandal et al., 2017; Ganj et al., 2019; Vatanpour and Sanadgol, 2020), and was also successfully applied to electron-beam-induced grafting of vinyl monomers by the Nasef group (Nasef et al., 2011; Nasef et al., 2012; Nasef et al., 2013; Nasef et al., 2014).

In this study, a highly efficient method for coupling the protein BSA to PVDF polymer membranes using EB irradiation was investigated. The aim was to develop a one-step approach providing high BSA grafting yields while reducing experimental costs, such as BSA amount, time, or irradiation dose, without compromising filtration performance. First, the process was characterized in terms of the amount and homogeneity of grafted BSA using a high number of nine process factors and a Minimum-Run Resolution V (MR5) design. Significant parameters were subsequently used to optimize the procedure applying the RSM approach. Models as well as optimized settings were confirmed, and modified membrane samples were extensively characterized, and discussed with respect to literature.

## 2 Materials and Methods

### 2.1 Materials

Commercially available polyvinylidene fluoride flat sheet microfiltration membrane (PVDF; ROTI^®^, 0.45 µm) was purchased from Carl Roth (Karlsruhe, Germany). Bovine serum albumin stock solution (BSA, mass fraction of ω = 30% in saline) was obtained from Sigma Aldrich (St. Louis, United States). Triton^®^ X-100 washing detergent and ethanol (EtOH, absolute) was purchased from Merck (Darmstadt, Germany). BSA quantification was performed using a commercial assay (Pierce^™^ BCA Protein Assay Kit) from Thermo Scientific (Rockford, IL, United States). Deionized water in Millipore^®^ quality was used for all steps. All chemicals were of analytical grade and used without further purification.

### 2.2 BSA Immobilization

#### 2.2.1 General Procedure

**FIGURE 1:**
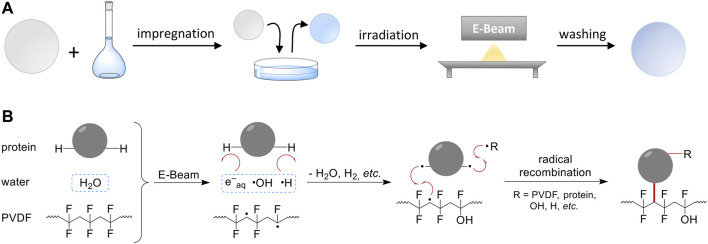
Scheme of the general method and proposed reaction mechanism. **(A)** The RIGI process solely utilizes a polymer membrane and an aqueous solution of the modifying compound (here: BSA). The membrane is impregnated with the aqueous solution. In case of hydrophobic polymers, a pre-wetting step has to be included. Finally, irradiation with electron beam and washing is performed. **(B)** Simplified scheme of the proposed reaction mechanism according to recent studies (Schmidt et al., 2021). Electron beam irradiation results in the formation of active species such as PVDF mid-chain radicals, and water radiolysis products, primarily solvated electrons, OH radicals, and H radicals. Due to its low abundance (ω ≤ 2%), the radiation chemistry of the protein is of minor importance. Immediately, water radiolysis products react with the solute, *e.g.*, *via* H abstraction, to form protein radicals. Finally, radical recombination reactions lead to covalent coupling between protein molecules and polymer chains.

The general procedure of this EB-induced graft immobilization as well as the proposed mechanism is given in Figure 1. By using the DoE approach, nine parameters (factors) were determined and systematically investigated within defined limits (factor levels). The first step was the impregnation of a PVDF membrane (Ø = 47 mm) with an aqueous solution of BSA. Subsequently, the impregnated membrane was removed from the impregnation solution and irradiated by EB. Irradiation was performed in N_2_ atmosphere with O_2_ quantities <15 ppm in a self-built low-energy electron accelerator. The voltage and conveyor speed were 160 kV and 2.1–3.0 m min^−1^, respectively. An irradiation dose was applied by adjusting the beam current from 0–20 mA. Directly after EB irradiation, a washing step at 450 rpm was performed to remove non-covalently attached molecules (10 min, ω = 0.5% Triton X-100, 4 × 10 min H_2_O in excess). Finally, the modified membrane samples were dried over night at 37°C and used for further characterization. The nine process factors are given in Table 1.

**TABLE 1:** Investigated nine process factors for the EB-based graft immobilization of BSA on PVDF.

Factor	Name	Description/Comment
A	BSA concentration	BSA solutions were freshly prepared each time from stock solution (*ω* = 30%, or *ß* ≈ 300 g L^−1^)
B	temperature of BSA	simulation of protein storage conditions by using tempered solvents for preparing BSA solutions
C	impregnation time	adsorption and potential enrichment of BSA on the PVDF polymer membrane
D	impregnation volume	amount of BSA solution added to the PVDF membrane located in a plastic Petri dish
E	irradiation dose	dose of the EB irradiation by adjusting the beam current of the electron accelerator
F	impregnation method (1-step)	addition of EtOH to the BSA solution in order to enable wettability of the hydrophobic membranes (a mass fraction of ω = 30% is at least necessary, as shown in Supplementary Figure S2 in SI)
G	impregnation method (2-steps)	hydrophobic nature of PVDF requires a pre-wetting step (1 min EtOH, 5 × 5 min H_2_O in excess to exchange EtOH) prior to the actual impregnation with aqueous BSA solution
H	shaking conditions	shaking of the Petri dish using a platform shaker (Vibramax 100, Heidolph, Schwabach, Germany)
J	surficial drying	irradiation of impregnated membranes either wet, or partially dried (by placing them in laboratory tissue and applying a roller to remove surficial liquid spots, which could affect homogeneity)

#### 2.2.2 MR5 Design

To reduce this large number of potential factors, first, a characterization of the EB process was performed in order to retain only the vital few ones. For this purpose, statistics software Design-Expert 13 (Stat-Ease, Minneapolis, MN, United States), and a Minimum-Run Resolution V (MR5) characterization design were applied (Table 2) (Whitcomb and Anderson, 2004). The MR5 design provides information on main effects, as well as two-factor interactions (2FI). By choosing this design, the number of experiments could be reduced from 512 (2^9^ for a full 2-level factorial design) to only 46 experimental runs. The responses examined were 1) the final immobilized BSA amount after washing (BSA grafting yield, GY) as the average of five measurement points (see characterization section); 2) the standard deviation (SD); and 3) the relative standard deviation (RSD) of the GY as a measure of homogeneity. Signal-to-noise ratio (S/N) values were derived from historical data and used to calculate power in Design-Expert. Power is a statistical measure for the ability of the design to detect significant effects (Supplementary Table S1 in SI). After collecting all response measurement data, the design was analyzed by using analysis of variance (ANOVA) for each response. The model was reduced by selecting only terms with a p-value ≤ 0.01, *i.e.*, highly significant terms, in order to reduce the number of model terms. Eventually, the final model is obtained according to Eq. 1 *via* multiple linear regression

$$y=\beta_{0}+\beta_{1}x_{1}+\beta_{2}x_{2}+\beta_{12}x_{1}x_{2}+\ldots +\varepsilon$$ (1)

where: *y* is the response; *x*_*i*_ is the process factor; β_*i*_ is the model parameter; and epsilon, is the random error or noise. Various diagnostics provided by Design-Expert were considered in order to statistically validate the model. Furthermore, the subsequent optimization *via* RSM is used to provide a more accurate model of the vital few factors. Finally, the model was confirmed by performing five additional runs for two sets (factor levels: all low; and all high) and comparing the average to calculated prediction intervals (PI).

**TABLE 2:** Investigated nine process factors by using a MR5 characterization design. Factors can be divided in numerical and categoric types (here: presence or absence, yes or no). Given are the factor simplified names (see Table 1), units, and each a low factor level (coded as –), and a high factor level (coded as +), respectively. The factor levels were chosen to cover a practical range of common settings of this EB processing method. The MR5 design resulted in 46 experimental runs.

Factor	Name	Units	Type	Low level (–)	High level (+)	Comment
A	BSA	g L^−1^	Numeric	1	10	Corresponds to ω = 0.1%, or ω = 1%
B	Temperature	°C	Numeric	10	20	Storage in refrigerator, or at room temperature
C	Time	min	Numeric	0.1	10	Impregnation time of 6 s, or 10 min
D	Volume	mL	Numeric	5	15	Impregnation volume
E	Dose	kGy	Numeric	20	200	Irradiation dose of EB
F	EtOH	y/n	Categoric	no	yes	Yes: impregnation solution with EtOH (*ω* = 30%)
G	Pre-wetting	y/n	Categoric	no	yes	Yes: prior pre-wetting step of pristine membrane
H	Shaking	y/n	Categoric	no	yes	Yes: shaking at 450 rpm on a platform shaker
J	Drying	y/n	Categoric	No	yes	Yes: surficial drying prior to EB irradiation

#### 2.2.3 RSM Design

The MR5-based characterization resulted in a reduced number of significant factors (see *results* section), which were used for optimization with RSM and Design-Expert 13. Initially, a Central Composite Design (CCD) was chosen. Center points were replicated six times to estimate the prediction capability, and 14 non-center points were performed including factorial points, as well as axial points to cover extreme factor combinations. The CCD was randomized, rotatable (k = 1.68179), and employed a reduced cubic model. Investigated factors included three numeric parameters, and a refined categoric factor for the overall impregnation method, *i.e.*, either utilizing only a pre-wetting step, or only EtOH addition instead of pre-wetting. Examined responses were the same as in the MR5 design (Supplementary Table S1 in SI). Upon evaluation, a significant lack-of-fit (LOF) test was observed, implying that there is a higher model that can better describe the data. For this reason, the initial CCD design was augmented resulting in additional 15 runs (7 for the model, three for LOF test, three replicates, and two additional points). The final RSM design was thus converted to an I-optimal design with a cubic model and a total of 55 experimental runs. Moreover, factor levels were adjusted to a lowest and a highest limit (Table 3). The design itself was evaluated using the fraction of design space (FDS) tool. An FDS score of 84% (S/N = 2), or 97% (S/N = 3), respectively, proved a high quality of the design to be used for optimization (Supplementary Figure S3 in SI). The data was analyzed using ANOVA and processed as described earlier. After a reduced model was obtained, optimized settings for specific criteria (see *results* section) were calculated using Design-Expert, and finally, confirmed by running five replicate runs for each optimized method.

**TABLE 3:** Investigated process factors of I-optimal RSM design. Given are the simplified names (see Table 1), units, and each a lowest, and highest factor level limit, respectively. Please note, the categoric factor ‘method’ was introduced as combination of the two previous factors describing possible impregnation methods, *i.e*., preceding pre-wetting step, or addition of EtOH to the impregnation solution.

Factor	Name	Units	Type	Lower limit	Upper limit
A	BSA	g L^−1^	numeric	1.0	18.4
B	time	min	numeric	0.1	12.4
C	dose	kGy	numeric	20	234
D	method	—	categoric	1-step (EtOH)	2-steps (pre-wetting)

### 2.3 Characterization

#### 2.3.1 Quantification of BSA Grafting Yield

After performing all experimental runs of a design, the amount of BSA grafted onto the membrane was calculated as the grafting yield (GY; mg m^−2^) employing

$$GY=\frac{w_{g}}{A}$$ (2)

where *w*_*g*_ is the weight of the protein grafted onto the membrane, and A is the surface area. The GY as main response was determined by using a commercial assay based on bicinchoninic acid (BCA, Pierce protein assay) (Smith et al., 1985). Briefly, five specimens of Ø = 10 mm were stamped out from each dried PVDF sample (Ø = 47 mm, 1x center, 4x off-center, see Supplementary Figure S4A in SI), placed into a 48-well microplate, and were incubated with 300 µL of BCA reagent for 30 min at 300 rpm in an oven (37°C). Subsequently, 200 µL of the reacted solution was transferred to a 96-well microplate within 5 min and measured at 562 nm in a microplate reader (Infinite^®^ M200, Tecan, Groedig, Austria). The average (BSA GY, response R1), the standard deviation (SD, response R2), and the relative standard deviation (RSD, response R3) were calculated (*n* = 5). Calibration curves were generated using BSA standards from the assay kit. It should be noted that due to the hydrophobic nature of PVDF, some corrections must be applied: Calibration must be performed in the presence of pristine membrane samples to account for adsorption of the dye. In addition, there was a distinct difference if the membrane was pre-wetted before addition of the BSA standard since more protein will be distributed within the inner membrane area and thus, might be less accessible to the BCA reagent. In the case of process characterization (MR5), however, there were samples that were not pre-wetted at all, so that two separate calibration curves were created (Supplementary Figure S4B in SI).

The chemical composition of pristine and modified PVDF membranes was investigated by X-ray photoelectron spectroscopy (XPS, AXIS Ultra, Kratos Analytical, Manchester, UK) with a monochromatic Al K_α_ source operated at 150 W (15 kV and 10 mA). Charge compensation and correction was applied to record survey spectra (160 eV pass energy, 1.0 eV resolution), and high-resolution spectra (40 eV pass energy, 0.1 eV resolution), respectively. Five spots were measured and averaged (total: *n* = 10; center: *n* = 2; off-center: *n* = 8; see Supplementary Figure S4A in SI). Since the only source for elemental N is expected to be the BSA macromolecule, the nitrogen atomic percentage on the grafted membrane surface measured by XPS (A_mN_) can be used to calculate the degree of BSA surface coverage (Γ_BSA_) on the membrane surface according to Eq. 3 (Zhang et al., 2013): Under the condition of complete coverage of the membrane surface with BSA, a nitrogen atomic percentage of A_pN_ = 12.95% is assumed, based on the total atomic composition in pure BSA powder.

$$\Gamma_{BSA}=\frac{A_{mN}}{A_{pN}}\cdot 100\%$$ (3)

#### 2.3.2 Polymer Characterization

Modifications of the polymeric material were investigated for modified samples and for pristine PVDF reference in terms of surface wettability, functional groups, and zeta potential. Surface wettability of membranes with water, as well as diiodomethane, CH_2_I_2_, was determined using a static contact angle measuring system (DSA30E, KRÜSS, Hamburg, Germany), applying the sessile drop method and the Young-Laplace model. Surface free energy was determined using the Owens-Wendt-Rabel-Kaelble model (Owens and Wendt, 1969). To reduce the influence of porosity, the samples were additionally compressed with high pressure (*p* = 200 kN) using a hydraulic press. Measurements were performed at least in triplicate. BSA grafting was further confirmed with Fourier-transform infrared spectroscopy (FTIR) taken in ATR (attenuated total reflection) mode and as transmission spectra using the Vector 22 FTIR spectrometer (Bruker, Billerica, United States), and a diamond cell (Golden Gate Specac). Spectra were recorded in the range of 4,000–500 cm^−1^ at a spectral resolution of 2 cm^−1^. Finally, zeta potential was determined by measuring the streaming potential in an adjustable gap cell of the SurPASS system (Anton Paar, Graz, Austria) utilizing 1 mM KCl solution. Four measurements were recorded for each pH step.

#### 2.3.3 Membrane Characterization

Membrane samples were analyzed in terms of morphology, porosimetry, mechanical properties, and water permeance according to methods described elsewhere (Schulze et al., 2013). Morphology was investigated by scanning electron microscopy (SEM, Ultra 55, Carl Zeiss Microscopy, Oberkochen, Germany). Magnifications ranged from 25- to 25,000-fold. Samples were cut manually and then coated with a thin (ca. 20 nm) chromium layer using the Z400 sputtering system (Leybold, Hanau, Germany). Pore size distribution and porosity were investigated in duplicate with a mercury intrusion porosimeter (PoreMaster 30, Quantachrome Instruments, Odelzhausen, Germany). Mechanical properties of membrane samples (Young’s modulus, E, and tensile strength, F_r_; *n* = 3) were determined using a tensile testing machine (inspekt mini 3 kN, Hegewald & Peschke, Nossen, Germany). Pure water permeance *P* was determined for samples (Ø = 47 mm, active area A = 13.3 cm^2^ considering the sealing ring) from the measured permeation time *t* for filtration of *V* = 200 ml water at a pressure of *p* = 1 bar using a stainless-steel filtration cell (16249, Sartorius Stedim, Göttingen, Germany). Permeance was measured in triplicate and calculated using Eq. 4

$$P=\frac{V}{A\cdot t\cdot p}$$ (4)

## 3 Results and Discussion

### 3.1 MR5 Design

Grafting of BSA on PVDF microfiltration flat sheet membranes was achieved by an EB process (Figure 1). EB irradiation is able to activate the polymer and the solutes by generating reactive species that can immediately react with each other (Schmidt et al., 2021). To estimate individual effect sizes and two-factor interactions (2FI), a Design of Experiments approach was used applying a MR5 design integrated in statistics software Design-Expert 13. By setting a low and a high factor level for each parameter, the factor space was largely covered. The investigated main response was the final BSA grafting yield, which was determined with a protein quantification assay (BCA). Furthermore, the (relative) standard deviation, (R)SD, as a measure of graft homogeneity was also investigated. The MR5 design, *i.e.*, combinations of factor levels and measured responses, is given in SI (Supplementary Table S2). Recently, a reaction mechanism for this radiation-induced graft immobilization (RIGI) was proposed based on computational and experimental studies of two small model molecules, glycine and taurine (Schmidt et al., 2021): Briefly, EB irradiation leads to 1) direct ionization of the organic polymer, subsequently forming reactive radical species; and 2) water radiolysis with the formation of reactive intermediates such as OH radicals, solvated electrons, or H radicals, respectively. Water radiolysis species are able to transfer dissolved compounds into radicals, *e.g.*, *via* H abstraction reaction. Thus, radical recombination reactions between the polymer surface and solutes result in the formation of covalent bonds facilitated by close contact due to, *e.g.*, hydrophobic or ionic interaction. We expect the same to be applied for protein immobilization, however, due to much larger size of these biomolecules, multiple covalent bonds per protein molecule could be formed facilitating the coupling process.

The MR5 design was analyzed *via* ANOVA. Initially, the model contained a total of 45 model terms, consisting of nine main effects (ME) and 36 two-factor interactions (2FI). The model was reduced manually with a criterion of p-value ≤ 0.01, in order to highly reduce the number of terms by retaining only the most significant ones. The final model included 10 model terms, consisting of 5 ME and five 2FI (Supplementary Table S3 in SI). The model F-value of 32.98 implies the model is significant, and that there is only a chance of *p* = 0.01% this F-value could occur due to noise. Furthermore, quality of the model was confirmed by a high adjusted correlation coefficient of R^2^_adj._ = 0.8766, indicating a strong correlation. Only three numerical factors (*i.e.*, BSA concentration, A; impregnation time, C; and irradiation dose, E), as well as two categorial factors (EtOH addition, F; and pre-wetting step, G) are statistically significant and thus, important. Please note, although the p-value of factor F (EtOH, *p* = 0.2356) was above the boundary criterion, the term must be included for hierarchical reasons because FG (EtOH ∙ pre-wetting) is highly significant. All numerical factors interact with each other (*i.e.*, AC, AE, and CE, respectively), as do the categorical ones (EF, and FG). Interestingly, neither temperature, nor impregnation volume, nor shaking, nor surficial drying showed a highly significant effect on BSA GY, indicating that the process can be simplified, and savings are possible (*e.g.*, lower impregnation volume). The final model is given in SI (Supplementary Table S4), however, since this MR5 design is not intended for predictions but rather for process characterization, the RSM model should be used instead.

Since the GY was calculated as average of five spots for each membrane sample (*i.e.*, experimental run), the standard deviation is a good approximation for the macroscopic homogeneity. In MR5 design, a factor space as large as possible is covered. This resulted in several factor combinations which are not suitable for the final process, however, are necessary to estimate effect sizes. Scatterplots revealed this issue, as low yields occurred more frequently (Supplementary Figure S5A in SI). This is mainly explained by low factor levels such as a BSA mass concentration of ß = 1 g L^−1^, or a dose of D = 20 kGy [lower amounts of generated reactive species (Buxton et al., 1988)]. Furthermore, the wettability of the hydrophobic membrane with aqueous BSA solution is crucial, which can be ensured either by the addition of ω ≥ 30% EtOH, and/or by performing a pre-wetting step. Moreover, due to characteristics of the BCA quantification assay, higher GY tend to produce higher SD. However, the opposite is true for RSD, which can usually reach very high values for low GY [Supplementary Figure S5B in SI, Horwitz-like relationship (Albert and Horwitz, 1997)]. For instance, run 11 resulted in a BSA grafting yield of 9.0 mg m^−2^ with SD = 9.5 mg m^−2^ and RSD = 105.1%, while run 21 resulted in a BSA grafting yield of 837.5 mg m^−2^ with SD = 21.8 mg m^−2^ and RSD = 2.6%. In consequence, this means that both responses, SD and RSD, would lead to a distorted representation in this special case. Nevertheless, since in the final process a high BSA GY is desirable, the RSD appears to be a good indicator for homogeneity. ANOVA analysis of RSD is shown in Supplementary Table S5 in SI, exhibiting fewer model terms than ANOVA results of SD (Supplementary Table S6 in SI). For RSD, a square root transformation had to be applied, and model reduction *via* p-values resulted in 13 model terms, consisting of seven main effects and six 2FI. Again, impregnation volume was not significant at all, however, impregnation time seemed to have no significant influence either. The model F-value of 14.81 implies the model is significant, and that there is only a chance of *p* = 0.01% this F-value could occur due to noise. A correlation coefficient of R^2^_adj._ = 0.7996 indicated a strong correlation.

**FIGURE 2:**
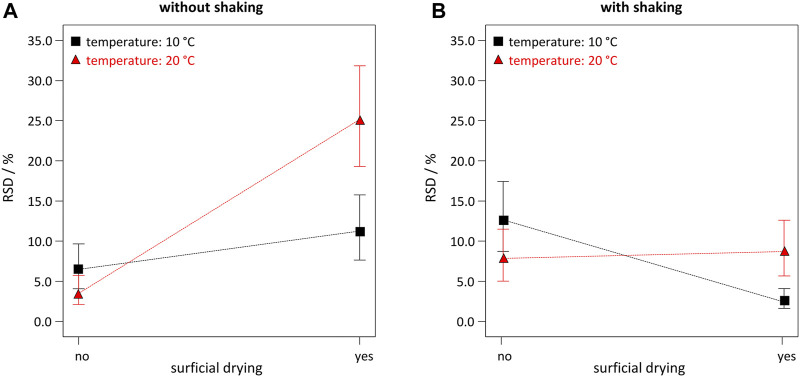
Interaction plots for correlations between RSD and the 2FI BJ (temperature ∙ drying). Plots were obtained for following settings: β_BSA_ = 10 g L^−1^, t = 10 min, D = 200 kGy, no EtOH, with pre-wetting step, and **(A)** without shaking; or **(B)** with shaking.

In contrast to BSA grafting yield, three additional main factors (temperature, B; shaking, H; and surficial drying, J) appeared in the model. However, all three showed high p-values of *p* = 0.2514, *p* = 0.3701, and *p* = 0.1667, respectively. Again, for hierarchical reasons these terms had to be included due to highly significant 2FI, *e.g.,* HJ (shaking ∙ drying, *p* = 0.0002), or BJ (temperature ∙ drying, *p* = 0.0024). Best settings were chosen for the less relevant factors by exploring interaction plots (Figure 2). Interaction plots revealed non-trivial two-factor interactions, which would be very hard to determine with the traditional OFAT approach. As a low RSD is desired, either a combination of no shaking, no drying, and room temperature/cooled solvents (Figure 2A), or a combination of shaking, drying and cooled solvents (Figure 2B) has to be applied.

In summary, the most important factor was the BSA concentration during impregnation since with increasing concentration the GY tended to increase as well, and the RSD decreased. For small organic molecules like glycine and taurine, it was found that a mass fraction of ω = 0.1% reached much better graft yields than higher or lower concentrations (Schulze et al., 2010; Schulze et al., 2012; Schmidt et al., 2021). However, in case of protein immobilization, this local optimum does not appear to be at 0.1% (1 g L^−1^) but rather much higher (≥10 g L^−1^). One reason could be the size of these biomolecules, which provide a larger attack surface for water radiolysis products and could in principle lead to formation of several reactive sites per molecule. On the one hand, this could promote multiple covalent bonds between the protein and the polymer, but on the other hand, it could also lead to cross-linking of protein molecules, which has not yet been resolved. Furthermore, the GY tended to increase with irradiation dose since with increased deposited energy, the amount of radical species tend to increase as well (Samuel and Magee, 1953; Buxton et al., 1988). Moreover, a longer impregnation time led to higher grafting yields, but significant amounts of immobilized BSA could already be detected after 6 s of impregnation. Interestingly, impregnation time was not important for RSD and thus, homogeneity. Finally, for all responses the impregnation methods were of significant importance, allowing for the use of both, the commonly applied 2-steps and the improved 1-step method. Please note, the impregnation method is only relevant for hydrophobic polymers such as PVDF, but for hydrophilic polymers like cellulose, neither a pre-wetting step, nor addition of EtOH must be performed. Other process parameters such as solution temperature, surficial drying, or shaking were only relevant for homogeneity and could be set to best settings and thus, kept stationary for follow-up experiments. The impregnation volume showed no influence at all. Hence, in potential technological implementations, advanced impregnation processes such as spraying instead of dip coating might be feasible. For each response, a model was created in Design-Expert, and confirmed with five replicated runs for two different settings (factor levels 1) all low; and 2) all high; see Table 2). Results confirmed the MR5 models with the only exception of RSD at low factor levels (Table 4). The models are further refined by the RSM design.

**TABLE 4:** Confirmation of response models (MR5 design). Two settings were examined (factor levels all low, and all high), and the data mean was compared to the predicted mean and the 95% prediction interval (PI). Responses SD and RSD had to be transformed using a square root function indicated as (sqrt).

Location	Analysis	Predicted mean	SD	*n*	95% PI low	Data mean	95% PI high
all low (–)	BSA GY	41.6	112.3	5	−114.7	7.5	198.0
	SD (sqrt)	2.8	1.9	5	0.4	2.3	6.2
	RSD (sqrt)	64.9	20.2	5	38.5	*33.1*	94.1
all high (+)	BSA GY	885.0	112.3	5	737.8	914.6	1,032.2
	SD (sqrt)	14.6	4.5	5	7.6	21.1	23.0
	RSD (sqrt)	12.4	8.6	5	1.6	2.3	28.3

### 3.2 RSM Design

The RSM design, *i.e.*, combinations of factor levels and measured responses, is given in SI (Supplementary Table S7). According to results gained by MR5 design, the following best settings were used for ease of operation: impregnation volume V = 7 ml, temperature T = 20°C, without shaking, and without drying, respectively. Response R1, BSA GY, was analyzed *via* ANOVA and the model was reduced manually with a criterion of p-value ≤ 0.05. The final model consisted of 17 terms, including (expectedly) all main effects, two-factor interactions, as well as quadratic and cubic terms (Table 5). The model F-value of 79.87 implies the model is significant, and that there is only a chance of *p* = 0.01% this F-value could occur due to noise. Furthermore, the quality of the model was confirmed by a very high adjusted correlation coefficient of R^2^_adj._ = 0.9620, indicating a strong correlation. Moreover, excellent prediction capability was proven by a very high prediction coefficient of R^2^_pred._ = 0.9349. A LOF test F-value of 1.26 implies that the LOF is not significant relative to the pure error, *i.e.*, the model is suitable to describe the data, and there is no need to use higher order models.

**TABLE 5:** ANOVA results for response R1, BSA grafting yield (RSM, I-optimal design).

Source	Sum of squares	Df	Mean square	F-value	p-value
Block	2.092‧10^6^	1	2.092‧10^6^	—	—
Model	4.649‧10^6^	17	2.735‧10^5^	79.87	<0.0001
A: BSA	3.896‧10^5^	1	3.896‧10^5^	113.77	<0.0001
B: time	16,620.00	1	16,620.00	4.85	0.0341
C: dose	14,523.90	1	14,523.90	4.24	0.0467
D: method	88,863.16	1	88,863.16	25.95	<0.0001
AC	2.902‧10^5^	1	2.902‧10^5^	84.76	<0.0001
AD	1858.84	1	1858.84	0.543	0.4660
BC	2,415.71	1	2,415.71	0.706	0.4065
CD	89,819.51	1	89,819.51	26.23	<0.0001
A^2^	2.764‧10^5^	1	2.764‧10^5^	80.72	<0.0001
B^2^	38,577.94	1	38,577.94	11.27	0.0019
C^2^	2.043‧10^5^	1	2.043‧10^5^	59.67	<0.0001
ACD	32,371.55	1	32,371.55	9.45	0.0040
AC^2^	1.663‧10^5^	1	1.663‧10^5^	48.56	<0.0001
B^2^C	53,124.52	1	53,124.52	15.51	0.0004
C^2^D	77,938.43	1	77,938.43	22.76	<0.0001
A³	50,031.45	1	50,031.45	14.61	0.0005
C³	1.477‧10^5^	1	1.477‧10^5^	43.14	<0.0001
Residual	1.233‧10^5^	36	3,424.35		
Lack of Fit (LOF)	85,167.13	23	3,702.92	1.26	0.3380
Pure Error	38,109.51	13	2,931.50	—	—
Cor Total	6.865‧10^6^	54	—	—	—

**FIGURE 3:**
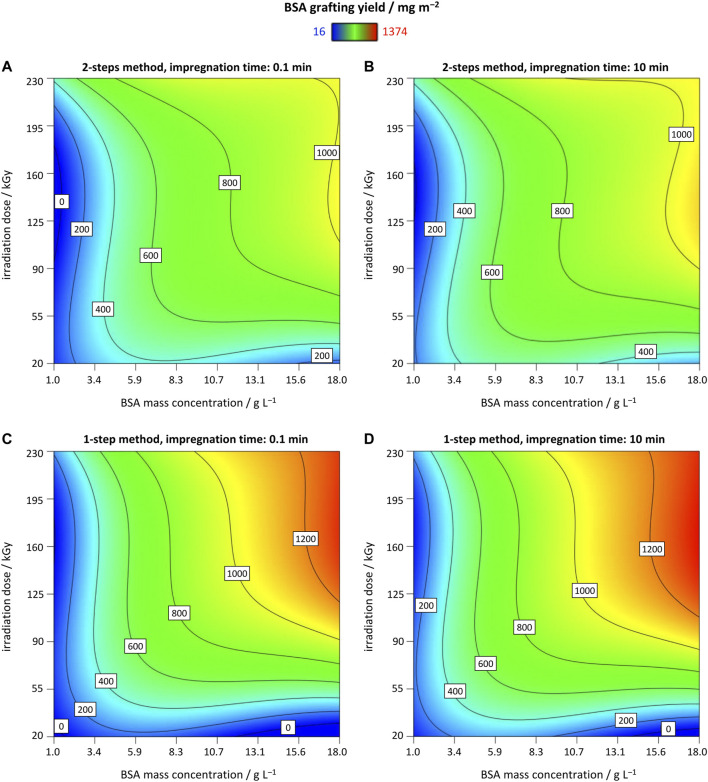
Contour plots of response R1, BSA GY, as a function of BSA mass concentration in the impregnation solution, irradiation dose, and impregnation time, t, for 2-steps method at **(A)** t = 0.1 min; **(B)** t = 10 min; and for 1-step method at **(C)** t = 0.1 min; **(D)** t = 10 min.

In comparison to the MR5 approach, scatterplots revealed that most data points are located at higher graft yields due to elimination of inappropriate settings, *e.g.*, lack of membrane wettability (Supplementary Figure S6A in SI). Unfortunately, both SD and RSD could not be analyzed *via* this RSM design because a significant LOF was detected. However, by inspecting the data scatterplots, most values were close to or below RSD = 5%, possibly as a consequence of the selected best settings exposed *via* MR5 characterization (Supplementary Figure S6B in SI). In general, higher BSA concentration and higher EB irradiation doses led to higher yields for both, 2-step method (Figures 3A,B), and 1-step method (Figures 3C,D), respectively. Interestingly, the 1-step method produced higher BSA grafting yields in average, which is unexpected at first since the addition of EtOH should dramatically decrease the concentration of water radiolysis products such as OH radicals due to scavenging. However, in contrast to typical OH scavengers like *tert*-butanol which produces relatively stable radicals due to sterically hindrance (Bobrowski, 2017), EtOH scavenging may result in more reactive radical species leading to further reaction cascades. Thus, the highest measured grafting yield of 1,374 mg m^−2^ ± 131 mg m^−2^ was achieved in run 16, applying 18.4 g L^−1^ BSA, 6.5 min impregnation using the 1-step method, and 150 kGy irradiation dose. Very high doses could lead to effects such as degradation of the polymer material. Since this RIGI approach utilizes aqueous impregnated membranes, the energy deposition is strongly attenuated by water molecules and thus, the membrane mechanic features are widely not affected (see analytic section). For the BSA concentration, the contour plots showed that a plateau of BSA GY is reached when using the 2-step impregnation method, but a slightly increasing trend for the 1-step method was observable. Nonetheless, further investigation has to be performed to exclude protein cross-linking. The final model for the 2-step method is given in Eq. 5, and for the 1-step method in Eq. 6, respectively.

$$2‐steps\;method:BSA\;GY=-90.9+110.9\cdot BSA+101.7\cdot time+3.4\cdot dose+1.1\cdot BSA\cdot dose-0.6\cdot time\cdot dose-12.5\cdot BSA^{2}-9.0\cdot time^{2}-0.07\cdot dose^{2}-0.004\cdot BSA\cdot dose^{2}+0.06\cdot time^{2}\cdot dose+0.3\cdot BSA^{3}+0.0003\cdot dose³$$ (5)

$$1‐step\;method:BSA\;GY=-307.6+90.5\cdot BSA+101.7\cdot time+8.8\cdot dose+1.4\cdot BSA\cdot dose-0.6\cdot time\cdot dose-12.5\cdot BSA^{2}-9.0\cdot time^{2}-0.1\cdot dose^{2}-0.004\cdot BSA\cdot dose^{2}+0.06\cdot time^{2}\cdot dose+0.3\cdot BSA^{3}+0.0003\cdot dose³$$ (6)

**FIGURE 4:**
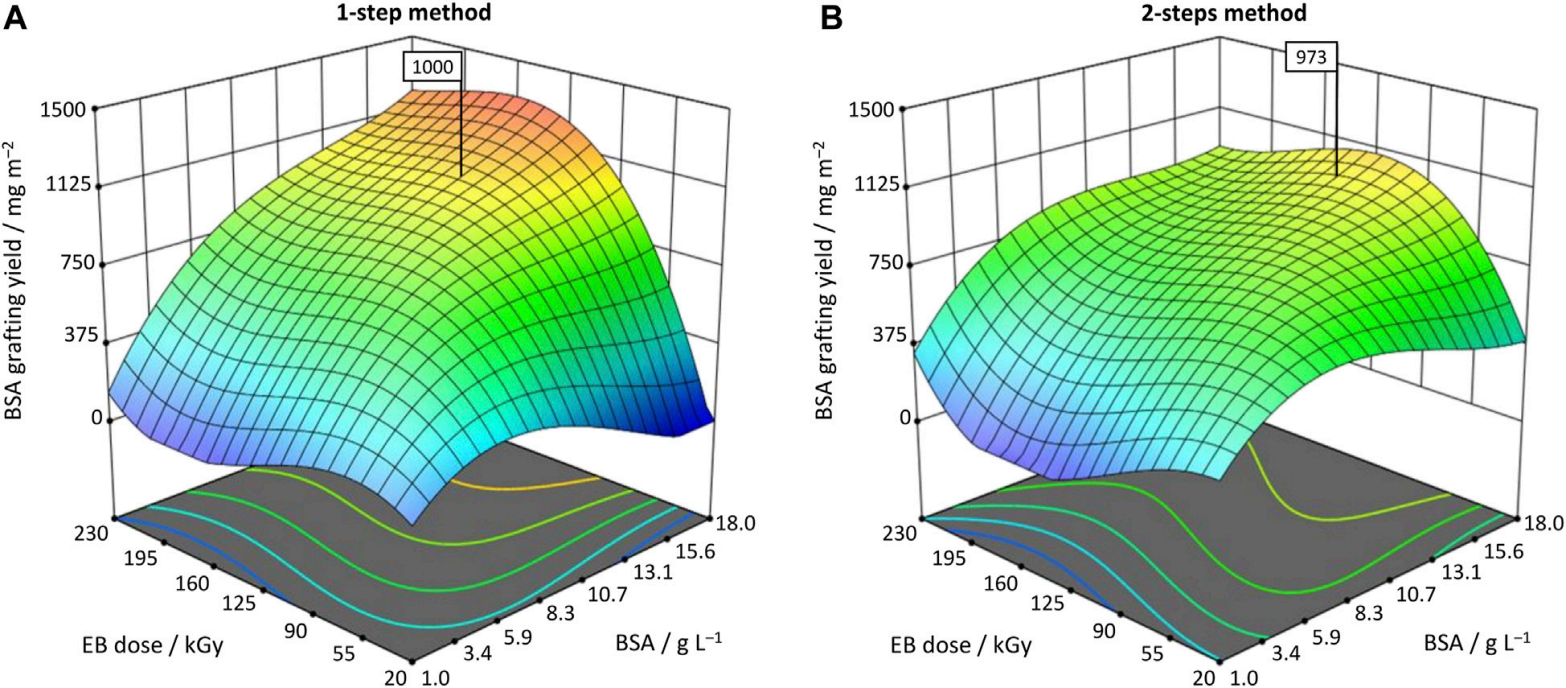
3D response surface plots for **(A)** optimized 1-step method (t = 2.7 min); and **(B)** optimized 2-steps method (t = 5.7 min). The flags highlight the predicted BSA grafting yield after numerical optimization *via* desirability function.

A high BSA grafting yield was targeted for this RSM. But simply determining the maximum value (1,374 mg m^−2^) might not always be useful, as side effects such as pore clogging are not considered. Hence, other responses such as filtration performance should be included, and an optimization *via* desirability function could be performed. Desirability is a mathematical method to find an optimum and one of the most widely used methods for analyzing multiple responses (Derringer and Suich, 2018). For this study, an optimization *via* desirability was carried out using statistics software Design-Expert 13. The GY was set at a target value of 1,000 ± 50 mg m^−2^, and at the same time the aim was to minimize the BSA mass concentration, impregnation time, and irradiation dose, respectively (Figure 4). For the 1-step method, the optimization predicted a BSA mass concentration of β_opt._ = 11.5 g L^−1^, an impregnation time of t_opt._ = 2.7 min, and a dose of D_opt._ = 117 kGy. For the 2-steps method, predicted optimal settings were β_opt._ = 14.5 g L^−1^, t_opt._ = 5.7 min, and D_opt._ = 107 kGy. Interestingly, the newly introduced 1-step method was predicted to achieve similar results with lower BSA concentration and impregnation time, reducing overall complexity and cost. Only the dose had to be set slightly higher (*Δ* = 10 kGy), but this is too low to be practically relevant. Again, the models were confirmed by performing five replicates for each location. In both methods, the BSA GY mean was within the 95% prediction interval and thus, confirmed the models (Table 6). Data revealed an average BSA GY of 955 mg m^−2^ ± 3.6% for the 1-step method, and 993 mg m^−2^ ± 5.6% for the 2-steps method, respectively. Covalency could be confirmed indirectly by comparing the adsorption reference, *i.e.*, employing the same conditions but 0 kGy: 41 mg m^−2^ ± 56% for the 1-step method, and 63 mg m^−2^ ± 17% for the 2-steps method, respectively.

**TABLE 6:** Confirmation of response models (RSM, I-optimal design). Two settings were examined (optimum for 1-step, and 2-steps method). The data mean was compared to the predicted mean and the 95% prediction interval (PI). Responses SD and RSD could not be analyzed since no model could be established (see text).

Location	Analysis	Predicted mean	SD	*n*	95% PI low	Data mean	95% PI high
1-step: β_BSA_ = 11.5 g L^−1^, t = 2.7 min, D = 117 kGy	BSA GY	998.9	58.5	5	932.2	954.9	1,065.7
	SD	Not analyzed		5	—	33.9	—
	RSD	Not analyzed		5	—	3.6	—
2-steps: β_BSA_ = 14.5 g L^−1^, t = 5.7 min, D = 107 kGy	BSA GY	972.6	58.5	5	889.4	993.0	1,055.9
	SD	Not analyzed		5	—	55.1	—
	RSD	Not analyzed		5	—	5.6	—

Finally, the reusability of the impregnation solution was investigated. A major advantage of the RIGI approach for surface modification of polymer-based membranes is the omission of coupling chemicals. Since chemically induced coupling reactions are typically carried out in batch operation, the reaction solution can usually only be used once. Especially in the case of protein immobilization, chemically induced crosslinking can occur due to the presence of numerous functional groups. Instead, in the RIGI process the reaction solution is used only for impregnation; no contamination occurs. Reusability was demonstrated by impregnating each a fresh PVDF membrane five times consecutively in the same solution (optimized 1-step conditions). Subsequently, EB irradiation and washing were performed as usual. Results for each repetition were as followed, given in mg m^−2^: **1**) 971 ± 28; **2**) 946 ± 29; **3**) 976 ± 31; **4**) 968 ± 26; and **5**) 951 ± 28. Considering the standard deviation, it was found that first, the EtOH content (*ω* = 30%) was sufficient to allow multiple wetting steps. Second, about 100% of the initial GY was reproduced in each repetition proving the extraordinarily high efficiency of this method. Further studies on a continuous scale are needed to characterize the limitations of reusability.

### 3.3 Characterization of Modified Membranes

All characterizations were performed using samples produced *via* the optimized 1-step method. First, XPS confirmed the covalent coupling of BSA since the N content increased from 0 to 10.1% ± 0.7%, as well as the S content raised from 0 to 0.6% ± 0.1% (top side, *n* = 10, Table 7). In the center, the N content reached up to 11.0 ± 0.2% (*n* = 2), and off-center the average N amount was 9.9 ± 0.6% (*n* = 8), see Supplementary Table S8 in SI. By comparing the elemental ratio F/C, it could be shown that a BSA layer had been immobilized on the membrane surface since the ratio decreased from 85.2% (PVDF-Ref) to 13.1% (PVDF-*g*-BSA). Since the average N amount was found to be 10.1%, an average BSA surface coverage of Γ_BSA_ = 78.3% according to Eq. 3 was calculated. For N_max_ = 11.1%, the maximum BSA surface coverage was determined to be Γ_BSA_ = 85.9%. In Table 8, grafting results of this study were compared to literature data. It could be shown that the optimized grafting yield of this study is up to ten times higher than other works. To our knowledge, the highest BSA grafting yield of this study (1,374 mg m^−2^) is the highest reported GY so far. Interestingly, by calculating the GY per reaction time, the optimized 1-step method reached up to 230-fold higher efficiency (19,100 mg m^−2^ h^−1^) than the best literature procedure that could be found (83 mg m^−2^ h^−1^ *via* 500 mg m^−2^ within 6 h reaction time (Fang et al., 2010). Thus, excellent efficiency and effectivity of this advanced immobilization method was demonstrated.

**TABLE 7:** XPS measurement data of pristine PVDF reference, and PVDF-*graft*-BSA modified *via* optimized 1-step method (*n* = 10).

Sample	Elemental composition/at%						Elemental ratio/%			
	C	F	O	N	S	Si^a^	F/C	O/C	N/C	S/C
PVDF-Ref, pristine	52.69	44.90	2.14	0.00	0.00	0.28	85.2	4.1	0.00	0.00
PVDF-*g*-BSA	65.08	8.47	15.32	10.14	0.55	0.44	13.06	23.53	15.59	0.84
	±1.11	±2.42	±1.01	±0.69	±0.09	±0.17	±3.92	±1.29	±1.08	±0.13

a Si appears to be an impurity from the pristine PVDF, membrane, e.g. from the manufacturing process.

**TABLE 8:** Comparison of grafting yields for immobilization of BSA on different polymers. In case of multiple reported data, highest yields were noted. Given are the applied BSA mass concentration, the reaction time (for RIGI: impregnation time), as well as three kinds of grafting yield parameters. Please note, literature GY data was converted from μg cm^−2^ to mg m^−2^ to faciliate comparison (conversion factor: 10).

Sample	BSA/g L^−1^	Time/h	GY/mg m^−2^	N/at%	Γ_BSA_/%	Ref
PVDF-*g*-BSA (RIGI, avg)^a^	11.5^b^	0.05	955	10.1	78.3	This study
PVDF-*g*-BSA (RIGI, max)^a^	18.4^b^	0.1	1,374	11.1	85.9	
PMMA-*g*-BSA	—	24	138	—	—	Kang et al. (1993)
PSf-*g*-PAA-BSA	10.0	2.5	93	—	—	Ulbricht and Riedel (1998)
PES/PANAA-*g*-BSA	10.0	7	309	9.1^c^	–	Fang et al. (2009)
PES/P (AN-VP-AA)-*g*-BSA	10.0	6	500	—	—	Fang et al. (2010)
PE/PDA-*g*-BSA	1.0	24	—	11.6^c^	—	Zhu et al. (2011)
PES/PVP-AA-*g*-BSA	10.0	7	87	—	—	Liu et al. (2012)
CPES-*g*-BSA	40.0	24	623	—	—	Wang et al. (2012)
PP_NWF_-*g*-PAA-BSA	1.0	12	—	9.1	70	Zhang et al. (2013)
PP-*g*-P (PEGMA-co-GMA)-BSA	10.0	72	—	2.5	19	Li et al. (2014)
PVDF-DAMP-*g*-BSA	1.0	24	1,280	—	—	Vitola et al. (2015)
PES/PDA-*g*-BSA	2.0	24	—	11.8^c^	—	Wu et al. (2018)

a Average of optimized 1-step method (avg), or highest determined value within this study (max), respectively.

b Reusability studies showed that the impregnation solution could be used multiple times to produce ≈100% of initial graft yield.

c Trunk polymer consists of N containing groups, which might contribute to the signal depending on BSA layer thickness.

**FIGURE 5:**
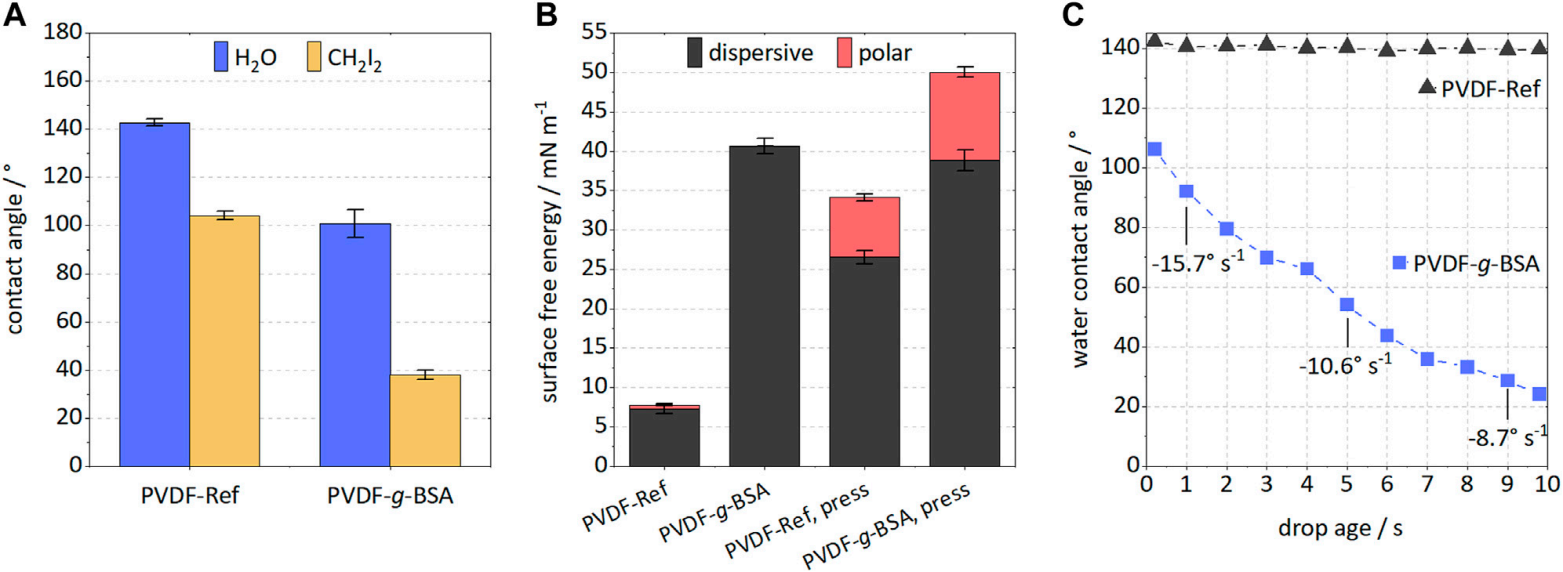
Investigation of membrane surface hydrophilicity for PVDF-Ref and PVDF-*g*-BSA (optimized 1-step method). **(A)** contact angle measurement using water and CH_2_I_2_; **(B)** surface free energy with polar and dispersive components for raw and pressed samples; and **(C)** dynamic attenuation curve of water contact angle with drop age for BSA-grafted sample (given are three decay rates of contact angle referred to the initial value). Please note, the reference showed no change in contact angle within 10 s.

Polymer characterizations revealed distinct improvements of the grafted samples. The static water contact angle decreased from 142.8° to 100.8° (Figure 5A). By using water and diiodomethane, respectively, surface free energies (SFE) could be calculated (Figure 5B). The BSA immobilization increased the overall SFE, rendering this modification to be promising for filtration applications or biomedical usage. Interestingly, mostly the dispersive part of the SFE raised corresponding to increased weak interactions. By minimizing effects of surface roughness and porosity *via* pressing the samples at high pressure, it was revealed that the polar part of SFE increased slightly. Since the polar SFE component corresponds to strong interactions due to localized shifts in electron density, a higher dispersive component could be more desirable for water filtration applications since electrostatically induced fouling might be reduced (Breite et al., 2015; Breite et al., 2016). Dynamic attenuation curves of water contact angles are often used as a measure of surface hydrophilicity to mitigate effects of surface roughness, capillary forces, or contraction in the dried state, respectively. A delayed water intake and thus, contact angle reduction, was monitored for the BSA grafted sample (Figure 5C). Within 10 s the contact angle decreased from 106° to 24°, suggesting that the hydrophilicity of PVDF membrane was improved significantly. An initial decay rate of −15.7°s^−1^ was determined, which, however, declines with time. An improved filtration performance can be expected due to stabilized long-term hydrophilization of the membrane surface.

**FIGURE 6:**
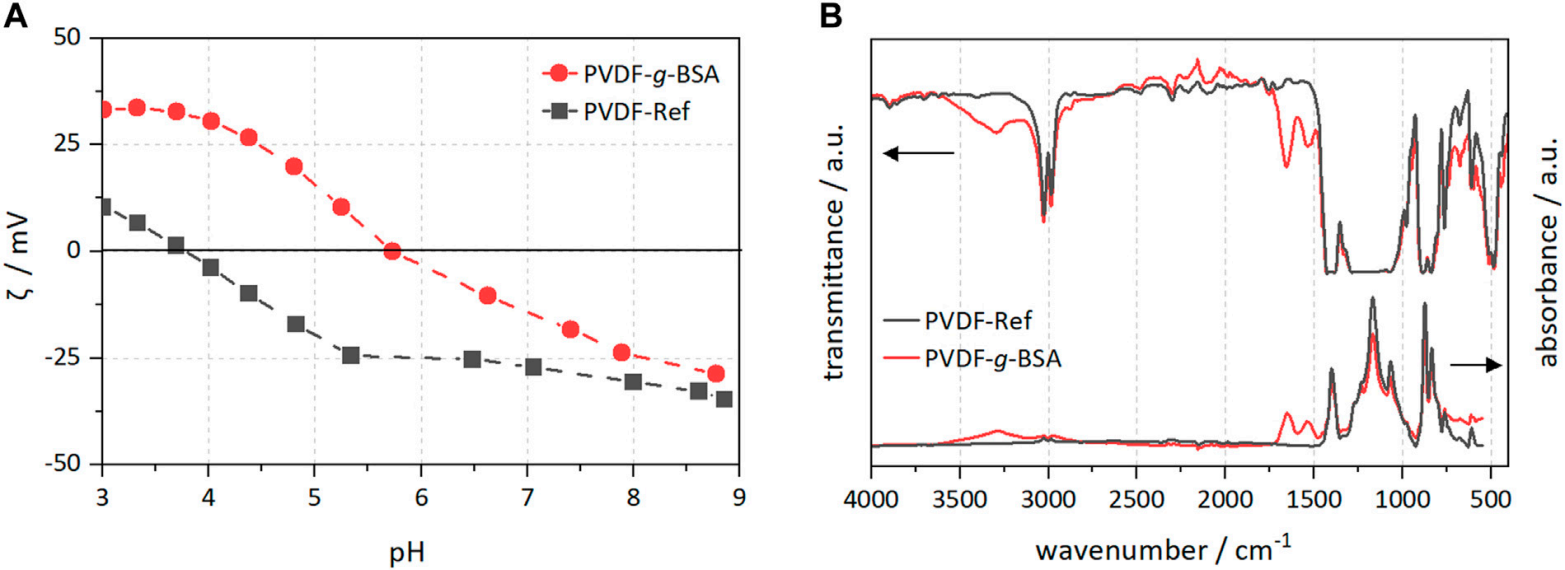
PVDF surface characterization of modified and reference samples. **(A)** Zeta potential measurements; and **(B)** FTIR spectra in transmission and ATR mode.

Zeta potential and FTIR measurements indicated alterations of the PVDF surface chemistry. There are changes in the overall zeta potential curves (Figure 6A), *e.g.*, at pH < 8 the zeta potential was shifted to higher values, and below pH 5.5, the zeta potential is even net positive. In addition, the isoelectric point (IEP) shifted from 3.78 to 5.74 (top side), and from 3.82 to 5.34 (bottom side), respectively. Thus, the surface charge became more positive overall as a result of BSA immobilization, and the zeta potential curves indicated an amphoteric behavior of the modified membranes. Pristine PVDF is known to have an acidic IEP due to adsorption of OH^−^ in water (Zimmermann et al., 2001). For the FTIR spectra of PVDF-*g*-BSA, the newly emerging peaks at 1,654 and 1,534 cm^−1^ indicate the presence of amide groups corresponding to the amide I (O=C-N-H) stretch vibrations and amide II (N-H) vibrations, which suggests that BSA was grafted successfully onto the surface of the PVDF membrane. Furthermore, a broad peak appeared at 3,300 cm^−1^ which was due to the N-H of the amino group in BSA (Figure 6B).

Investigations of mechanical membrane properties revealed that EB irradiation (hence, the RIGI process) does not impact the overall stability of the samples. It is known that ionizing radiation can induce degradation and cross-linking reactions within fluoropolymers (Lyons, 1995; Forsythe and Hill, 2000; Dargaville et al., 2003). However, since RIGI utilizes aqueous impregnated membranes, the deposited energy *via* irradiation might be better attenuated by the water molecules acting as a kind of degradation protection. Tensile testing was performed to evaluate Young’s modulus (E) and tensile strength (F_r_). PVDF-Ref yielded E = (165 ± 11) N mm^−2^ and F_r_ = (1.24 ± 0.04) N, while PVDF-*g*-BSA yielded E = (159 ± 13) N mm^−2^ and F_r_ = (1.33 ± 0.16) N, respectively. Thus, tensile testing confirmed that the mechanical properties were not affected by the irradiation-based immobilization. Mercury intrusion porosimetry showed similar porosity of 54.7 ± 5.0% *vs* 51.7 ± 1.5%, for PVDF-Ref *vs* PVDF-*g*-BSA, respectively. Mean pore of grafted membrane (0.68 ± 0.04 µm) was slightly higher than reference (0.53 ± 0.03 µm), however, this might be attributable to inhomogeneities in the employed membrane. Permeance examination of PVDF-Ref (21,452 ± 143 L m^−2^ h^−1^ bar^−1^) and PVDF-*g*-BSA (20,258 ± 841 L m^−2^ h^−1^ bar^−1^) showed only minimal reduction of permeance without practical relevance, *i.e.*, an excellent filtration performance is maintained. Finally, scanning electron microscopy (SEM) confirmed that no changes in morphology, as well as only minimal clogging occurred during the treatment (Supplementary Figures S7, S8 in SI).

## 4 Conclusion

In this study, the model protein BSA was covalently coupled on PVDF polymer flat sheet membranes using an advanced radiation-induced graft immobilization (RIGI) procedure. This method utilizes solely an aqueous impregnated membrane and electron beam (EB) irradiation in order to cleanly and directly obtain grafted polymer materials. No additional chemicals such as coupling reagents, photoinitiators, catalysts, or organic solvents are needed, rendering this processing strategy as overall environmentally-friendly, safe and thus “green.” Furthermore, this approach is technologically relevant due to its potential for simple scale-up and the possibility of a continuous mode of operation. By applying a statistical Design of Experiments (DoE) approach, nine process parameters were investigated in terms of their importance for achieving high grafting yields and homogeneity. Four factors are highly significant, *i.e.*, BSA concentration, impregnation method and time, as well as irradiation dose. Subsequently, a process optimization using the response surface methodology (RSM) was performed resulting in a mathematical model for both, a commonly used 2-steps method and an improved 1-step method. The latter combines the impregnation step with a pre-wetting step, which is necessary for hydrophobic membranes such as PVDF. For the 1-step method, a BSA GY of 955 mg m^−2^ ± 3.6% was already obtained for a BSA mass concentration of 11.5 g L^−1^, an impregnation time of 2.7 min, and a dose of 117 kGy. The average BSA surface coverage was 78.3%. Successful grafting was confirmed *via* BCA protein quantification, XPS, FTIR, contact angle and zeta potential measurements, respectively. Furthermore, since this immobilization method does not use additional chemicals, the impregnation solution can be reused multiple times, which was demonstrated to result in a very high efficiency of reproducing ≈100% grafting yield after five consecutive cycles. The highest determined BSA GY of this study (1,374 mg m^−2^) is the highest reported graft yield so far. Furthermore, this is accompanied by a 230-fold higher efficiency (19,100 mg m^−2^ h^−1^) than the best literature procedure which is 83 mg m^−2^ h^−1^ *via* 500 mg m^−2^ in 6 h reaction time. Thus, excellent efficiency and effectivity of this advanced immobilization method was demonstrated. These results indicate that the modified PVDF membranes can be used in biomedical applications as well as in water treatment applications. Finally, this study proofs that the RIGI procedure can be applied for immobilization of proteins, peptides, or other molecules having more biological relevance while using a cleaner, more efficient and cheaper production.

## Data Availability

The original contributions presented in the study are included in the article/Supplementary Material, further inquiries can be directed to the corresponding author.
