# Supplementary material for: Highly Efficient One-Step Protein Immobilization on Polymer Membranes Supported by Response Surface Methodology
Source: Front Chem. 2022 Jan 18;9:804698. doi: 10.3389/fchem.2021.804698 (PMC8804297; doi:10.3389/fchem.2021.804698)
Supplement: Supplementary file 1 [file DataSheet1.PDF]

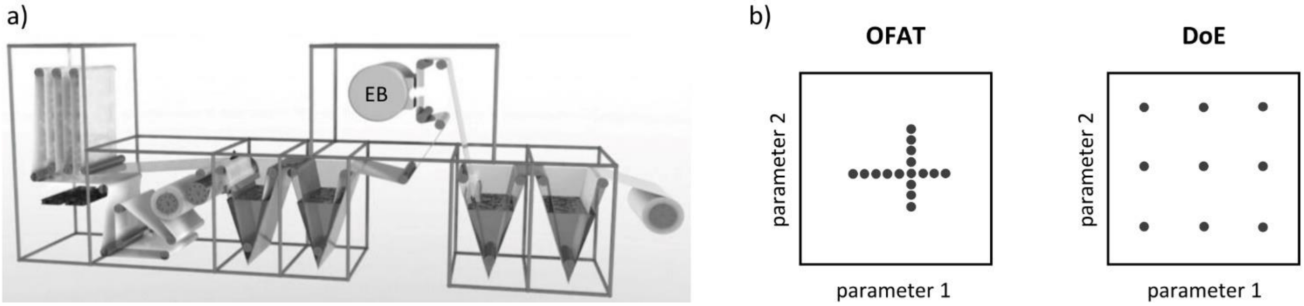

Supplementary Figure S1. (**a**) Visualization of a platform technology for continuous processing of polymer membranes by EB irradiation at the Leibniz Institute of Surface Engineering (IOM); (**b**) schematic comparison of the traditional experimental approach (one-factor-at-a-time, OFAT), and the Design of Experiments (DoE) approach. In DoE, all factors (i.e., parameters) are varied in parallel, resulting in systematically distributed points. After performing all experimental runs, a mathematical model is fitted to describe the data.

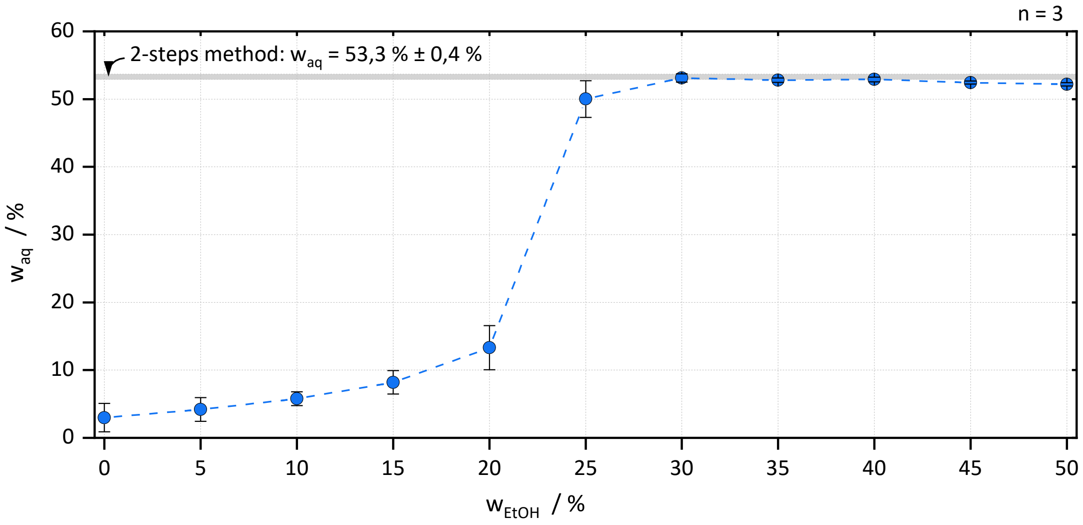

Supplementary Figure S2. Wetting of hydrophobic PVDF membranes. The mass fraction of the aqueous phase, w_{aq}, employing the 1-step impregnation method, depends on the mass fraction of EtOH, w_{EtOH}. Wetting has to be applied in order to obtain water-wetted hydrophobic membranes for subsequent modification. By adding at least 30% ethanol, the same amount of the aqueous phase could be achieved as when performing the regular 2-steps approach (*i.e.*, a separate pre-wetting step employing, first, impregnation in pure EtOH, and second, multiple exchange steps with pure water; grey bar).

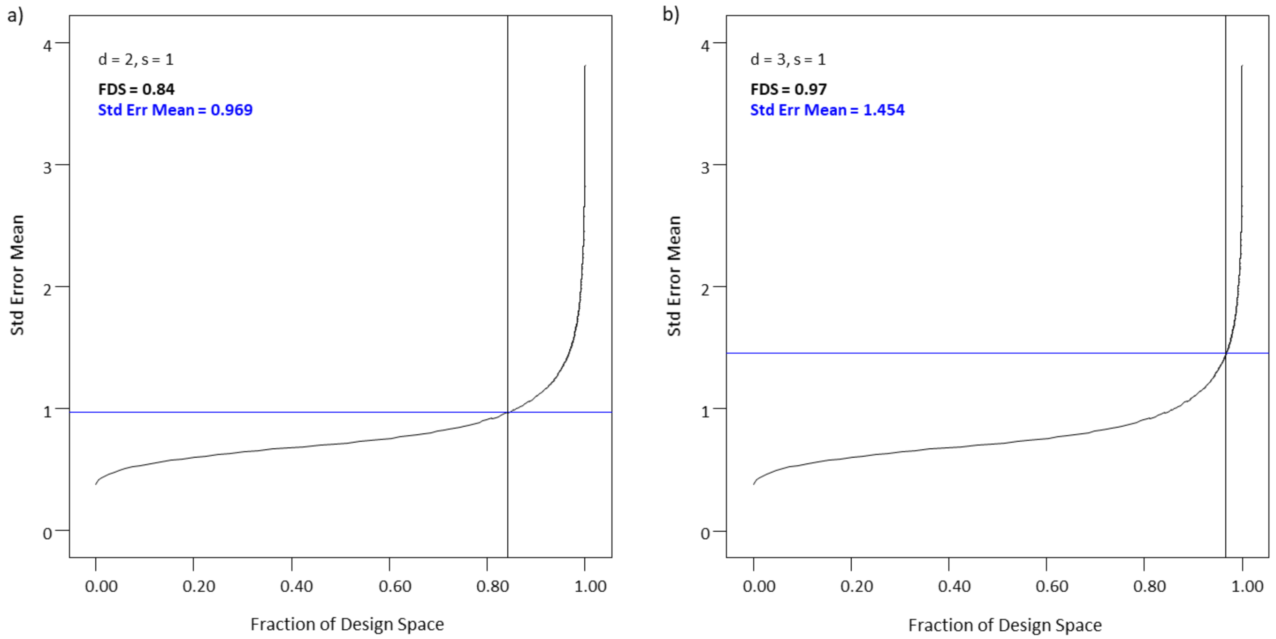

Supplementary Figure S3. FDS analysis of the RSM design. Optimization capability of the RSM design was supported by an FDS score of (**a**) 84% (S/N = 2); and especially, (**b**) 97% (S/N = 3).

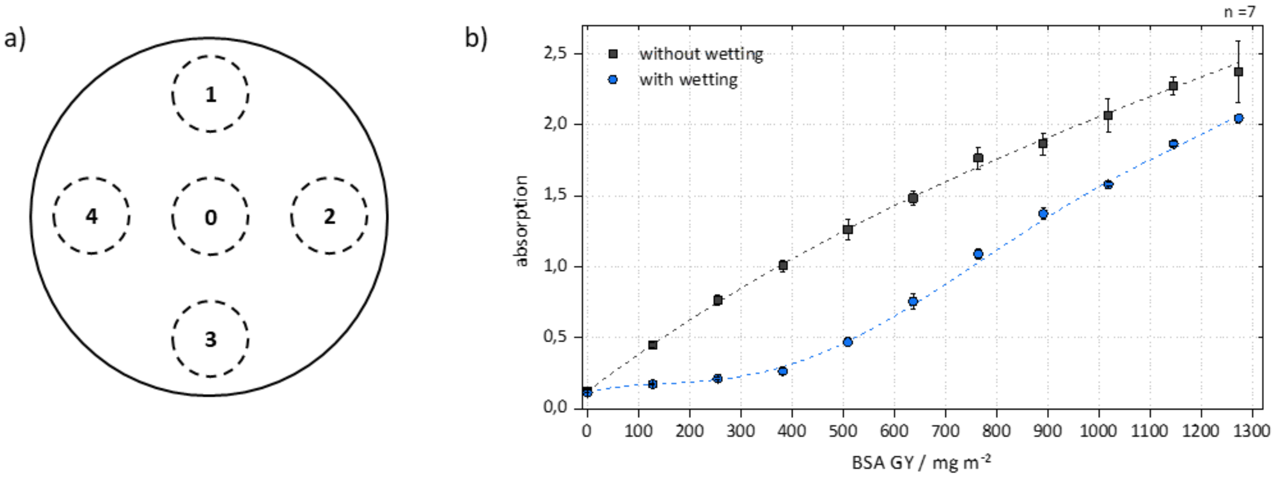

Supplementary Figure S4. Determination of BSA grafting yield, GY. BSA GY was calculated (**a**) as the average of five specimen (Ø = 10 mm) stamped out of a PVDF sample (Ø = 47 mm) at specific positions (center: spot 0; off-center: spot 1-4); and (**b**) employing two different calibration curves for either pre-wetted or non-wetted samples.

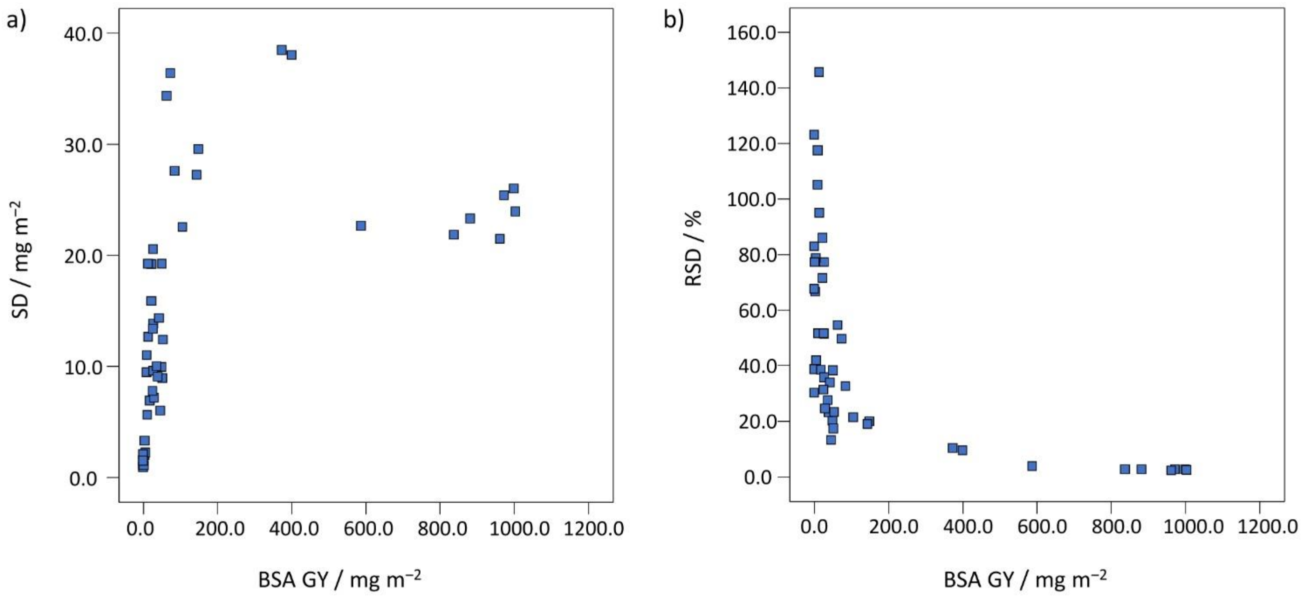

Supplementary Figure S5. Scatter plots of experimental data obtained via MR5 design, showing the correlations between BSA GY and (**a**) standard deviation, SD; or (**b**) relative standard deviation, RSD. The plots revealed that more runs yielded a low GY due to inappropriate factor combinations such as a too low BSA concentration, too low dose, or lack of wettability.

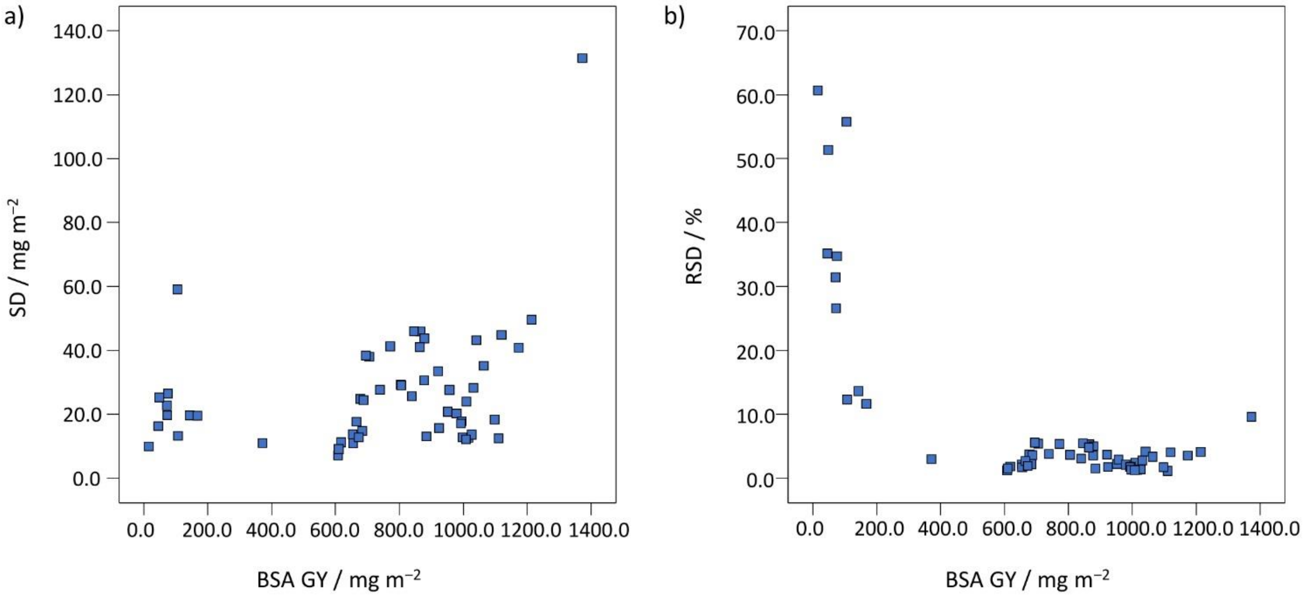

Supplementary Figure S6. Scatter plots of experimental data obtained via RSM design, showing the correlations between BSA GY and (**a**) standard deviation, SD; or (**b**) relative standard deviation, RSD.

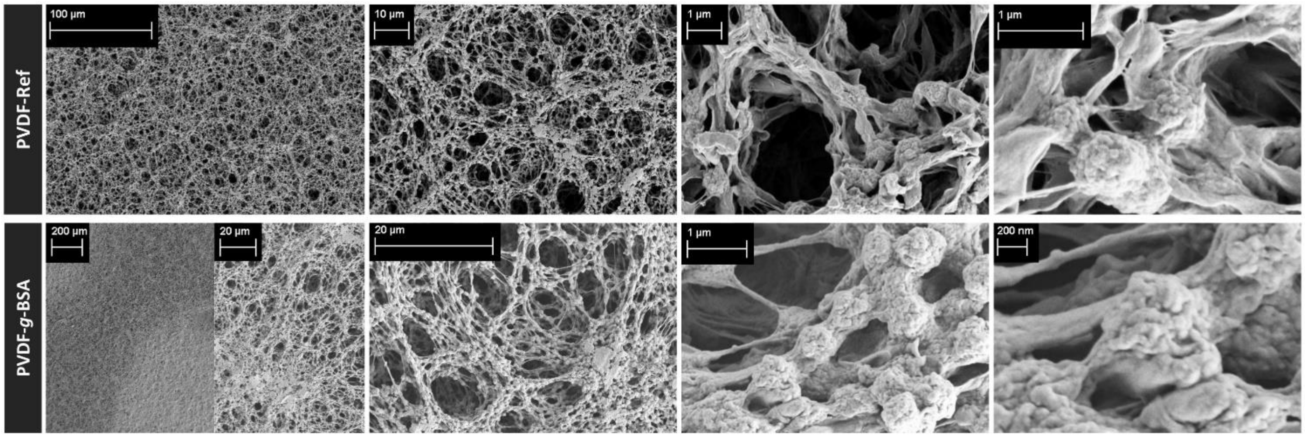

Supplementary Figure S7. SEM images of pristine PVDF-Ref and PVDF-*g*-BSA for the top side. Magnifications ranged from 25- to 25,000-fold.

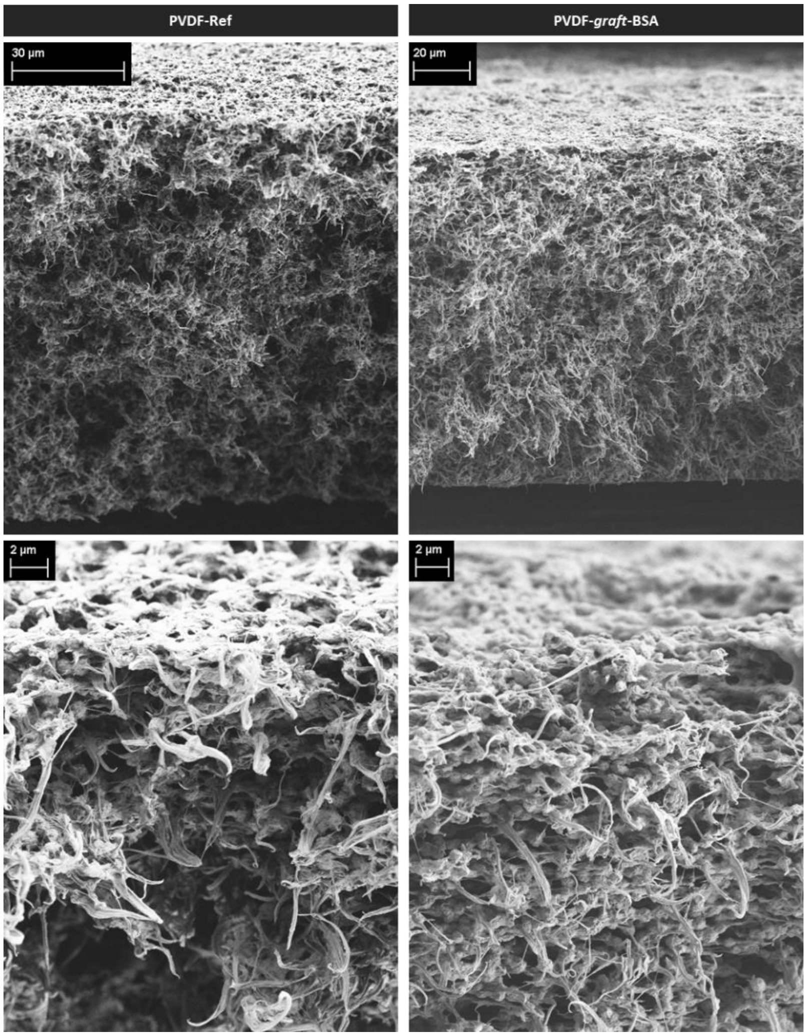

Supplementary Figure S8. SEM images of membrane cross sections.

Supplementary Table 1. Examined responses. Given are the simplified names (see text), units, and data regarding expected signals and noise (from historical data). Furthermore, the power for the main effects was calculated to be 99.9% for this MR5 design, confirming a very high statistical ability to detect significant effects.

Response	Name	Units	Signal	Noise	S/N	Power (ME)
R1	BSA GY	mg m^{-2}	50	25	2	99.9%
R2	SD	mg m^{-2}	4	2	2	99.9%
R3	RSD	%	1	0.5	2	99.9%

Supplementary Table S2. MR5 design of nine factors (F), and measured responses (R).

	**F1**	**F2**	**F3**	**F4**	**F5**	**F6**	**F7**	**F8**	**F9**	**R1**	**R2**	**R3**
**#**	**BSA** **g L^{-1}**	**temp.** **°C**	**time** **min**	**volume** **mL**	**dosage** **kGy**	**EtOH** **y/n**	**pre-wetting** **y/n**	**shaking** **y/n**	**drying** **y/n**	**BSA GY** **mg m^{-2}**	**SD** **mg m^{-2}**	**RSD** **%**
1	10	20	0.1	5	200	no	no	no	yes	0.0	1.6	82.9
2	10	10	0.1	5	20	no	no	yes	yes	0.0	1.1	30.3
3	10	20	10	15	200	yes	no	no	no	1003.4	23.9	2.4
4	1	10	10	15	20	yes	yes	no	no	50.3	19.2	38.3
5	10	20	0.1	15	200	no	no	yes	yes	0.0	0.9	38.7
6	10	20	0.1	5	200	no	no	yes	no	84.5	27.6	32.6
7	10	10	10	15	200	yes	yes	no	no	961.8	21.5	2.2
8	1	20	10	15	200	no	no	yes	no	22.2	15.9	71.5
9	1	10	10	15	200	yes	yes	yes	yes	25.9	13.4	51.6
10	10	10	10	5	200	no	yes	yes	yes	972.7	25.4	2.6
11	1	20	0.1	15	20	yes	yes	yes	no	9.0	9.5	105.1
12	1	20	10	15	20	yes	no	yes	yes	9.4	11.0	117.5
13	10	10	0.1	5	20	no	yes	yes	no	400.0	38.0	9.5
14	1	10	0.1	5	200	yes	yes	yes	no	13.3	12.7	94.9
15	1	10	0.1	5	200	yes	no	yes	yes	22.4	19.2	86.0
16	1	20	0.1	5	200	yes	no	no	no	105.4	22.6	21.4
17	1	10	10	15	200	no	no	no	yes	0.0	1.5	67.6
18	1	10	0.1	15	200	yes	yes	no	yes	10.9	5.6	51.6
19	1	10	0.1	5	20	no	no	no	no	1.4	1.1	77.2
20	1	20	0.1	5	20	yes	yes	no	yes	13.2	19.2	145.6
21	10	20	10	5	200	yes	yes	no	yes	837.5	21.8	2.6
22	1	20	10	15	20	no	yes	yes	yes	36.4	10.0	27.6
23	10	20	10	5	20	yes	yes	yes	no	24.9	7.8	31.3
24	10	20	0.1	5	20	yes	no	yes	yes	45.6	6.0	13.2
25	10	10	10	5	20	no	no	no	no	26.8	9.6	35.7
26	10	10	0.1	15	20	no	yes	no	yes	373.5	38.5	10.3

27	10	10	0.1	15	20	yes	no	no	yes	39.3	9.1	23.1
28	1	10	0.1	15	200	no	yes	yes	no	143.7	27.2	19.0
29	10	10	0.1	15	200	no	no	no	no	49.2	9.9	20.2
30	1	10	10	5	200	no	yes	no	no	53.4	12.4	23.2
31	1	20	10	5	200	no	no	yes	yes	4.2	3.3	78.7
32	10	10	10	15	200	yes	no	yes	yes	999.4	26.0	2.6
33	1	10	10	5	20	no	no	yes	no	3.0	2.0	66.6
34	1	20	0.1	5	200	no	yes	yes	yes	29.2	7.2	24.6
35	1	20	0.1	15	20	no	no	no	yes	0.0	2.1	123.1
36	10	20	10	15	20	yes	yes	no	yes	73.4	36.4	49.6
37	10	20	0.1	5	20	yes	yes	no	no	51.5	8.9	17.3
38	10	10	0.1	15	20	yes	no	yes	no	26.6	20.6	77.3
39	10	20	10	15	200	no	yes	no	no	881.9	23.3	2.6
40	1	10	0.1	15	20	yes	yes	yes	yes	18.0	6.9	38.4
41	10	20	10	15	20	no	no	yes	no	42.4	14.3	33.8
42	10	20	10	5	20	no	yes	no	yes	148.7	29.5	19.9
43	1	10	10	5	20	yes	yes	no	yes	63.0	34.4	54.5
44	1	10	10	5	200	yes	no	no	no	26.9	13.8	51.4
45	1	20	10	5	20	no	no	no	no	5.4	2.2	41.9
46	10	20	0.1	15	200	yes	yes	yes	no	587.2	22.6	3.9

Supplementary Table S3. ANOVA results for response R1, BSA grafting yield (MR5 design).

Source	Sum of Squares	df	Mean Square	F-value	p-value
Model	$4.157 \cdot 10^6$	10	$4.157 \cdot 10^5$	32.98	< 0.0001
A: BSA	$9.856 \cdot 10^5$	1	$9.856 \cdot 10^5$	78.19	< 0.0001
C: time	$2.498 \cdot 10^5$	1	$2.498 \cdot 10^5$	19.82	< 0.0001
E: dose	$5.586 \cdot 10^5$	1	$5.586 \cdot 10^5$	44.31	< 0.0001
F: EtOH	18358.56	1	18358.56	1.46	0.2356
G: pre-wetting	$1.763 \cdot 10^5$	1	$1.763 \cdot 10^5$	13.99	0.0007
AC	$1.109 \cdot 10^5$	1	$1.109 \cdot 10^5$	8.80	0.0054
AE	$7.374 \cdot 10^5$	1	$7.374 \cdot 10^5$	58.50	< 0.0001
CE	$3.585 \cdot 10^5$	1	$3.585 \cdot 10^5$	28.44	< 0.0001
EF	$1.889 \cdot 10^5$	1	$1.889 \cdot 10^5$	14.98	0.0005
FG	$2.852 \cdot 10^5$	1	$2.852 \cdot 10^5$	22.63	< 0.0001
Residual	$4.412 \cdot 10^5$	35	12605.20		
Cor Total	$4.598 \cdot 10^6$	45			

Supplementary Table S4. BSA GY models (MR5 design). Four models of response R1, BSA GY, were obtained as result from the MR5 design, depending on the utilization of pre-wetting or EtOH impregnation method. GY can be calculated using the three significant factors, *i.e.*, BSA mass concentration in g L^{-1}, impregnation time in min, and irradiation dosage in kGy.

**pre-wetting**	**no**	**yes**	**no**	**yes**
**EtOH**	**no**	**no**	**yes**	**yes**
β_0	+97.59819	+399.26927	+144.41530	+107.95932
BSA	-14.90085	-14.90085	-14.90085	-14.90085
time	-20.38883	-20.38883	-20.38883	-20.38883
dosage	-2.30846	-2.30846	-0.818919	-0.818919
BSA · time	+2.40342	+2.40342	+2.40342	+2.40342
BSA · dosage	+0.324302	+0.324302	+0.324302	+0.324302
time · dosage	+0.202399	+0.202399	+0.202399	+0.202399

Supplementary Table S5. ANOVA results for response R3, RSD (MR5 design).

Source	Sum of Squares	df	Mean Square	F-value	p-value
Model	307.28	13	23.64	14.81	< 0.0001
A: BSA	129.23	1	129.23	80.96	< 0.0001
B: temperature	2.18	1	2.18	1.36	0.2514
E: dose	11.42	1	11.42	7.15	0.0117
F: EtOH	5.10	1	5.10	3.19	0.0834
G: pre-wetting	50.00	1	50.00	31.33	< 0.0001
H: shaking	1.32	1	1.32	0.826	0.3701
J: drying	3.20	1	3.20	2.00	0.1667
AF	19.25	1	19.25	12.06	0.0015
BJ	17.27	1	17.27	10.82	0.0024
EF	9.27	1	9.27	5.81	0.0219
FG	16.99	1	16.99	10.64	0.0026
FH	8.44	1	8.44	5.29	0.0281
HJ	27.27	1	27.27	17.09	0.0002
Residual	51.08	32	1.60		
Cor Total	358.36	45			

Supplementary Table S6. ANOVA results for response R2, SD (MR5 design). A square root transformation of the data had to be applied. The model F-value of 13.61 implied the model is significant, and that there is only a chance of p = 0.01% this F-value could occur due to noise. A correlation coefficient of $R^2_{adj.} = 0.8486$ indicated a strong correlation.

Source	Sum of Squares	df	Mean Square	F-value	p-value
Model	94.83	20	4.74	13.61	< 0.0001
A: BSA	8.77	1	8.77	25.18	< 0.0001
B: temperature	0.84	1	0.84	2.41	0.1330
C: time	3.16	1	3.16	9.08	0.0058
D: volume	0.14	1	0.14	0.40	0.5308
E: dosage	0.12	1	0.12	0.33	0.5684
F: EtOH	3.07	1	3.07	8.82	0.0065
G: pre-wetting	10.06	1	10.06	28.885	< 0.0001
H: shaking	0.046	1	0.04	0.12	0.7269
J: drying	5.67	1	5.67	16.27	0.0005
BG	1.90	1	1.90	5.44	0.0280
CJ	2.62	1	2.62	7.52	0.0111
DE	2.40	1	2.40	6.90	0.0145
DJ	4.87	1	4.87	13.98	0.0010
EF	3.76	1	3.76	10.78	0.0030
EG	8.76	1	8.76	25.14	< 0.0001
EH	6.10	1	6.10	17.50	0.0003
EJ	1.87	1	1.87	5.37	0.0289
FG	12.58	1	12.58	36.11	< 0.0001
FJ	7.28	1	7.28	20.89	0.0001
HJ	3.12	1	3.12	8.97	0.0061
Residual	8.71	25	0.35		
Cor Total	103.54	45			

Supplementary Table S7. RSM design of most significant factors (F), and measured responses (R).

		F1	F2	F3	F4	R1	R2	R3
Block	Run	BSA<br>g L^{-1}	time<br>min	dosage<br>kGy	method<br>-	BSA GY<br>mg m^{-2}	SD<br>mg m^{-2}	RSD<br>%
Block 1	1	15,0	10,0	200	2-steps (pre-wetting)	998,3	12,7	1,3
Block 1	2	5,0	3,0	200	1-step (EtOH)	618,3	11,1	1,8
Block 1	3	10,0	6,5	150	2-steps (pre-wetting)	840,2	25,5	3,0
Block 1	4	15,0	3,0	200	1-step (EtOH)	1174,4	40,8	3,5
Block 1	5	10,0	6,5	150	1-step (EtOH)	979,5	20,2	2,1
Block 1	6	15,0	3,0	200	2-steps (pre-wetting)	993,6	17,0	1,7
Block 1	7	10,0	6,5	66	2-steps (pre-wetting)	805,2	29,2	3,6
Block 1	8	10,0	6,5	234	2-steps (pre-wetting)	879,1	43,6	5,0
Block 1	9	10,0	12,4	150	2-steps (pre-wetting)	866,4	45,8	5,3
Block 1	10	5,0	3,0	100	2-steps (pre-wetting)	609,3	7,0	1,1
Block 1	11	10,0	6,5	150	2-steps (pre-wetting)	846,0	45,9	5,4
Block 1	12	1,6	6,5	150	2-steps (pre-wetting)	74,0	19,6	26,5
Block 1	13	10,0	6,5	150	1-step (EtOH)	957,7	27,5	2,9
Block 1	14	5,0	10,0	200	1-step (EtOH)	695,7	38,3	5,5
Block 1	15	5,0	10,0	100	1-step (EtOH)	706,3	37,9	5,4
Block 1	16	18,4	6,5	150	1-step (EtOH)	1373,6	131,3	9,6
Block 1	17	10,0	6,5	150	1-step (EtOH)	1032,4	28,2	2,7
Block 1	18	10,0	6,5	66	1-step (EtOH)	878,4	30,5	3,5
Block 1	19	10,0	0,6	150	1-step (EtOH)	921,9	33,4	3,6
Block 1	20	10,0	6,5	150	1-step (EtOH)	1016,8	12,5	1,2
Block 1	21	10,0	0,6	150	2-steps (pre-wetting)	678,7	24,8	3,6
Block 1	22	10,0	6,5	150	2-steps (pre-wetting)	772,2	41,2	5,3
Block 1	23	15,0	10,0	100	1-step (EtOH)	1010,1	23,9	2,4
Block 1	24	18,4	6,5	150	2-steps (pre-wetting)	1064,7	35,1	3,3
Block 1	25	10,0	6,5	234	1-step (EtOH)	952,7	20,7	2,2
Block 1	26	10,0	6,5	150	1-step (EtOH)	885,6	13,0	1,5
Block 1	27	10,0	12,4	150	1-step (EtOH)	925,0	15,7	1,7
Block 1	28	10,0	6,5	150	2-steps (pre-wetting)	739,8	27,6	3,7
Block 1	29	5,0	10,0	200	2-steps (pre-wetting)	656,1	10,8	1,7
Block 1	30	15,0	10,0	200	1-step (EtOH)	1214,6	49,5	4,1
Block 1	31	1,6	6,5	150	1-step (EtOH)	107,7	13,2	12,3
Block 1	32	15,0	3,0	100	2-steps (pre-wetting)	996,6	17,8	1,8
Block 1	33	10,0	6,5	150	2-steps (pre-wetting)	806,6	29,0	3,6
Block 1	34	15,0	10,0	100	2-steps (pre-wetting)	1042,0	43,1	4,1
Block 1	35	5,0	10,0	100	2-steps (pre-wetting)	655,1	13,6	2,1
Block 1	36	15,0	3,0	100	1-step (EtOH)	1111,6	12,3	1,1
Block 1	37	5,0	3,0	200	2-steps (pre-wetting)	610,5	9,0	1,5

Block 1	38	5,0	3,0	100	1-step (EtOH)	684,4	14,6	2,1
Block 1	39	10,0	6,5	150	2-steps (pre-wetting)	864,4	40,9	4,7
Block 1	40	10,0	6,5	150	1-step (EtOH)	1098,5	18,3	1,7
Block 2	41	8,0	2,6	110	2-steps (pre-wetting)	673,8	12,7	1,9
Block 2	42	11,5	5,1	110	1-step (EtOH)	1008,9	12,0	1,2
Block 2	43	15,0	0,1	140	1-step (EtOH)	1120,4	44,8	4,0
Block 2	44	1,0	0,1	80	1-step (EtOH)	46,1	16,2	35,1
Block 2	45	8,0	2,6	110	2-steps (pre-wetting)	688,0	24,3	3,5
Block 2	46	1,0	5,1	20	1-step (EtOH)	144,2	19,5	13,6
Block 2	47	1,0	0,1	20	2-steps (pre-wetting)	76,1	26,4	34,7
Block 2	48	8,0	2,6	110	2-steps (pre-wetting)	665,9	17,6	2,6
Block 2	49	1,0	10,0	20	2-steps (pre-wetting)	105,9	59,0	55,7
Block 2	50	8,0	10,0	20	1-step (EtOH)	168,4	19,5	11,6
Block 2	51	8,0	0,1	20	2-steps (pre-wetting)	372,1	10,9	2,9
Block 2	52	10,3	0,1	20	1-step (EtOH)	16,2	9,8	60,6
Block 2	53	11,5	5,1	110	1-step (EtOH)	1027,3	13,5	1,3
Block 2	54	1,0	10,0	20	1-step (EtOH)	49,0	25,2	51,3
Block 2	55	15,0	5,1	20	1-step (EtOH)	72,3	22,7	31,4

Supplementary Table S8. XPS full data of PVDF-*g*-BSA measured at five spots.

sample	elemental composition / at%						elemental ratio / %			
	**C**	**F**	**O**	**N**	**S**	**Si**	**F/C**	**O/C**	**N/C**	**S/C**
PVDF-*g*-BSA, center (0, #1)	65.22	5.93	16.59	11.12	0.66	0.48	9.1	25.4	17.0	1.0
PVDF-*g*-BSA, center (0, #2)	65.95	5.35	16.63	10.83	0.58	0.67	8.1	25.2	16.4	0.9
PVDF-*g*-BSA, off-center (1, #1)	63.64	11.93	13.47	10.06	0.47	0.44	18.7	21.2	15.8	0.7
PVDF-*g*-BSA, off-center (1, #2)	63.74	11.21	14.21	9.99	0.54	0.30	17.6	22.3	15.7	0.8
PVDF-*g*-BSA, off-center (2, #1)	66.92	6.14	15.76	10.03	0.72	0.43	9.2	23.6	15.0	1.1
PVDF-*g*-BSA, off-center (2, #2)	65.25	8.30	15.43	9.83	0.49	0.71	12.7	23.6	15.1	0.8
PVDF-*g*-BSA, off-center (3, #1)	64.78	10.02	15.09	9.20	0.45	0.45	15.5	23.3	14.2	0.7
PVDF-*g*-BSA, off-center (3, #2)	65.29	6.72	15.97	11.12	0.61	0.30	10.3	24.5	17.0	0.9
PVDF-*g*-BSA, off-center (4, #1)	63.82	10.91	14.60	10.04	0.48	0.15	17.1	22.9	15.7	0.8
PVDF-*g*-BSA, off-center (4, #2)	66.20	8.14	15.47	9.20	0.48	0.50	12.3	23.4	13.9	0.7
PVDF-*g*-BSA, center: avg.	65.59	5.64	16.61	10.98	0.62	0.58	8.6	25.3	16.7	0.9
PVDF-*g*-BSA, center: SD	0.52	0.41	0.03	0.21	0.06	0.13	0.7	0.2	0.4	0.1
PVDF-*g*-BSA, off-center: avg.	64.96	9.17	15.00	9.93	0.53	0.41	14.2	23.1	15.3	0.8
PVDF-*g*-BSA, off-center: SD	1.20	2.16	0.85	0.60	0.09	0.17	3.6	1.0	1.0	0.1
